# Supplementary material for: Roles of ABCC1 and ABCC4 in Proliferation and Migration of Breast Cancer Cell Lines
Source: Int J Mol Sci. 2020 Oct 16;21(20):7664. doi: 10.3390/ijms21207664 (PMC7589126; doi:10.3390/ijms21207664)
Supplement: Supplementary file 1 [file ijms-21-07664-s001.pdf]

Roles of ABCC1 and ABCC4 in proliferation, migration and invasion of breast cancer cell lines

Floren G. Low, Kiran Shabir, James E. Brown, Roslyn M. Bill and Alice J. Rothnie*

College of Health & Life Sciences, Aston University, Aston Triangle, Birmingham, B4 7ET, UK

Supplementary Information

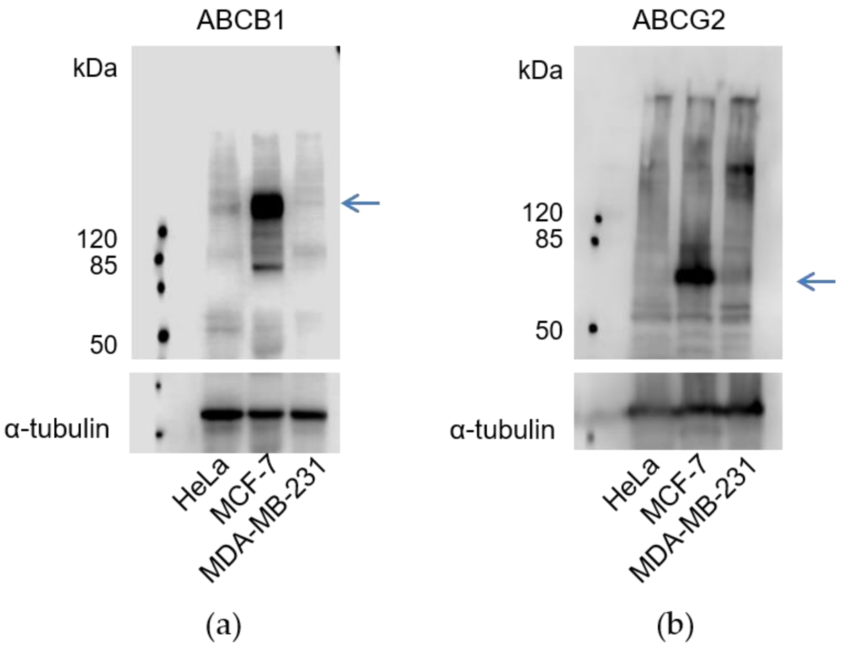

Figure S1. Expression of ABCB1/P-glycoprotein or ABCG2 in breast cancer cell lines. Membrane extracts (80μg protein/well) from MCF-7 and MDA-MB-231 cells were assayed by Western blot for expression of (a) ABCB1 or (b) ABCG2, alongside Hela cells as a positive control. The blue arrow indicates the band corresponding to ABCB1 (a) or ABCG2 (b). Comparable sample loading was monitored afterwards using the anti-tubulin primary antibody.

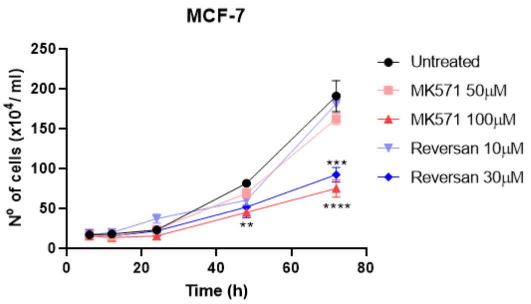

(a)

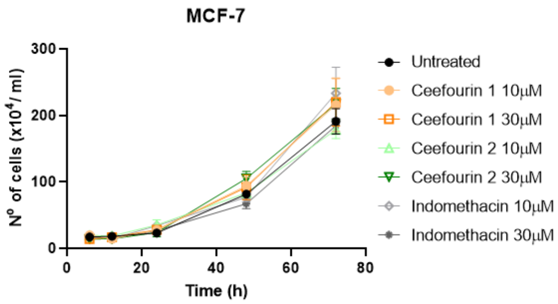

(b)

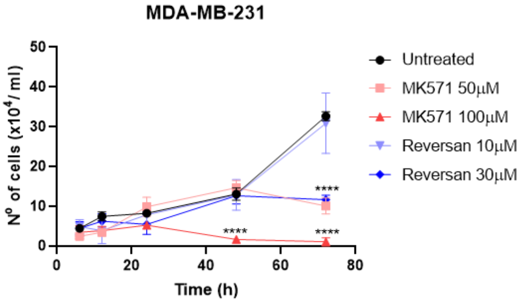

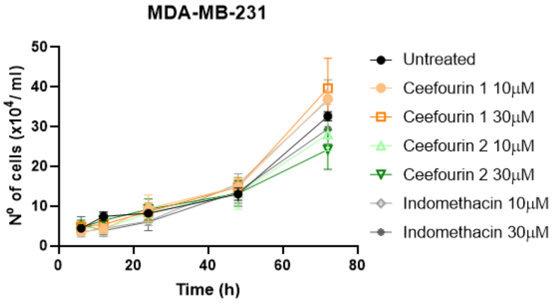

(c)

(d)

Figure S2. MK571 and Reversan affect the proliferation of breast cancer cells. 15000 MCF-7 cells (a & b) or 6000 MDA-MB-231 cells (c & d) were seeded in 24-well plates After 4 h of culture, cells were treated with inhibitors as detailed. At 6, 12, 24, 48 and 72 h after treatment cells were harvested, diluted in trypan blue at a ratio of 1:4, viewed using a haemocytometer and microscope and counted manually. Data are mean±SD, n≥5. Data were analysed using a two-way ANOVA with a Dunnett's post hoc test. $^{**}P<0.01$, $^{***}P < 0.001$, $^{****}P<0.0001$ significantly lower than the untreated sample.

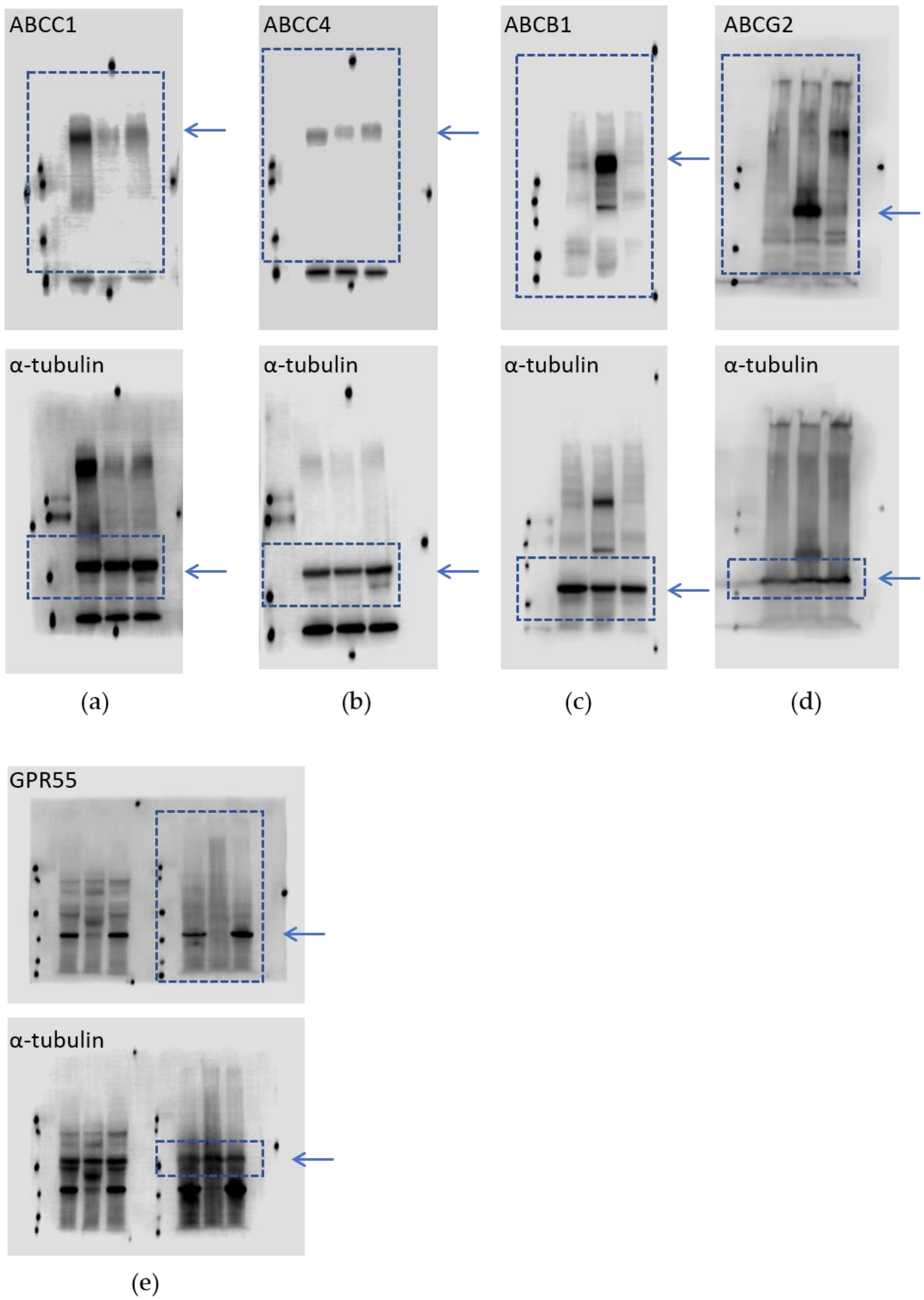

Figure S3. Original, uncropped Western blots for (a) Figure 1a, (b) Figure 1b, (c) Supplementary Figure 1a, (d) Supplementary Figure 1b, (e) Figure 8a. Dotted blue lines indicate the region that was cropped. Blue arrows indicate bands corresponding to the target protein.

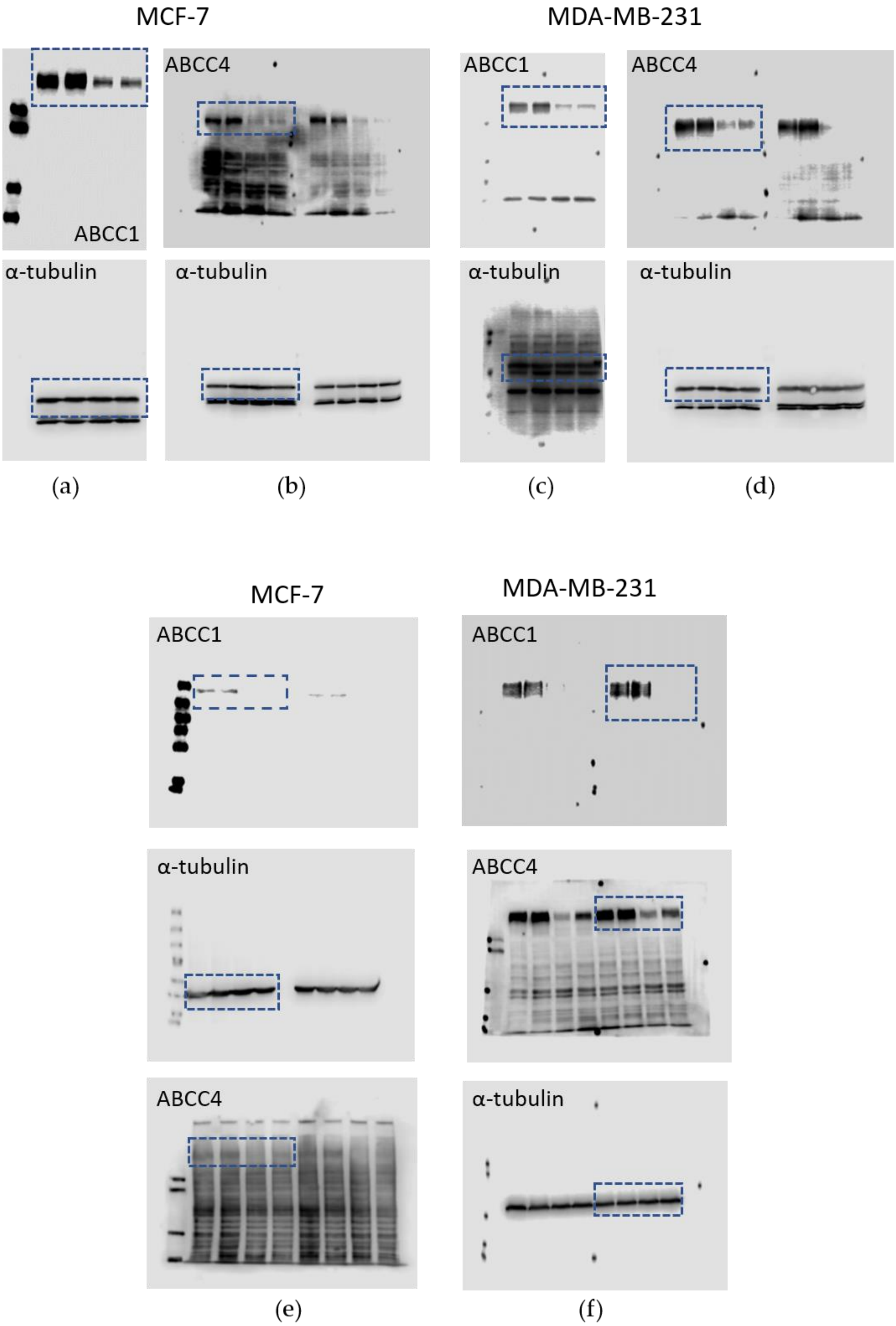

Figure S4. Original, uncropped Western blots for (a) Figure 5a, (b) Figure 5b, (c) Figure 5c, (d) Figure 5d, (e & f) Figure 5e. Dotted blue lines indicate the region that was cropped.
